# Sex, Gender and Gender Identity: A Case for Conceptual Clarity

**DOI:** 10.1111/apa.70686

**Published:** 2026-07-13

**Authors:** Mikael Landén

**Affiliations:** ^1^ Department of Psychiatry and Neurochemistry Institute of Neuroscience and Physiology, The Sahlgrenska Academy at the University of Gothenburg Gothenburg Sweden; ^2^ Department of Medical Epidemiology and Biostatistics Karolinska Institutet Stockholm Sweden

For most of the twentieth century, transsexualism—now more commonly termed gender dysphoria—was an extremely rare condition. When I compiled all applications for legal sex reassignment filed in Sweden between 1972 and 1992, the average was approximately twelve individuals per year [1]. Since then, the situation has changed dramatically. Beginning around 2013, referrals of children and adolescents to specialist gender services rose sharply, particularly among adolescent birth‐registered females. Comparable shifts in referral patterns have been reported across several other countries.

The clinical question is therefore no longer how health services should support a small group of adults with a rare condition, but how clinicians, researchers and policymakers should understand sex, identity and psychological distress among young people in an era shaped by social media, identity politics and rapidly changing social norms.

## 1 Conceptual Confusion

Current discussion of sex and gender is marked by considerable conceptual confusion. The terms *sex*, *gender* and *gender identity* are often used interchangeably, despite referring to different concepts. Biological sex is rooted in reproduction. Humans, like other animals, are organized to produce either small gametes (sperm) or large gametes (ova), which fuse at fertilisation to form a new individual who is male or female. In this biological sense, sex is binary.

Human traits are not binary, however. Sexual selection has produced average differences between the sexes in physical and psychological traits—men are on average taller than women, women on average less physically aggressive than men—but these traits overlap substantially between the sexes and are not part of the definition of sex itself. An unusually tall and aggressive woman is not thereby intermediate between the sexes. She remains unequivocally female—that is, an individual whose reproductive organisation is directed toward producing large gametes—and simply occupies one end of the normal range of height and aggression *within her own sex*. Traits that differ on average between males and females vary continuously within each sex, but this within‐sex variation does not make biological sex itself a spectrum.

Gender roles, by contrast, are cultural expectations about how men and women ought to behave. A girl whose interests align more closely with traditionally masculine gender roles, or a boy whose interests align with traditionally feminine ones, does not thereby become less female or male, or occupy some intermediate position on a sex spectrum.

The binary nature of biological sex places no constraint on how individuals lead their lives. Apart from reproduction, a man and a woman may be as similar or as different in their interests, personalities, appearance and life choices as any other two people. A risk arises, however, when interests and personalities are interpreted through the lens of rigid gender stereotypes. Individuals may come to perceive themselves as atypical and begin to question their sex rather than the stereotype itself.

The term gender identity was coined in the 1960s but did not enter widespread use until later. It is now routinely solicited in contexts ranging from healthcare questionnaires to consumer surveys. In a growing number of jurisdictions, legal gender recognition is now explicitly grounded in this concept. Sweden, for example, adopted such legislation in 2025. Despite its growing legal and clinical weight, however, gender identity remains poorly defined. Existing definitions typically describe it as a person's internal sense of gender without independently defining gender itself, making the concept difficult to operationalise and distinguish from related constructs [2].

For most people this conceptual ambiguity has little practical consequence. They know themselves to be male or female without experiencing this as a distinct identity. Nor did it matter greatly when gender dysphoria was a rare condition affecting a small and relatively homogeneous group of adults with a persistent conviction that they belonged to the opposite sex. It becomes considerably more consequential, however, when the poorly defined concept of gender identity is applied to a rapidly growing and clinically heterogeneous population of children and adolescents.

## 2 Gender Dysphoria as Distress, Not Only Identity

Clinically, gender dysphoria has traditionally been understood as a source of psychological distress rather than as a matter of identity per se. The diagnosis denotes the distress that can arise when a person's experience is incongruent with their sexed body. The concept emerged in the mid‐twentieth century as clinicians sought to understand and help individuals experiencing a persistent conflict between their bodies and their sense of self. Evidence that medical and surgical transition improves long‐term outcomes has always been limited, largely because controlled studies have not been feasible. Historically, this was not viewed as a pressing concern since treatment involved only a small number of well‐informed adults who themselves sought these interventions.

Today, the situation differs fundamentally. In place of a handful of adults each year, services are now seeing a rapidly growing number of children and adolescents. The reasons for this increase remain uncertain. Changing social norms, internet culture, increased access to gender care and increased media attention to transgender issues are all plausible contributors. A substantial proportion of young people referred for gender dysphoria present with co‐occurring psychiatric disorders or a history of trauma and some report that their gender dysphoria emerged relatively suddenly during adolescence. None of this implies that their distress is not genuine. It does, however, indicate that clinicians are now encountering a considerably more complex patient population than in the past, one poorly served by simple explanatory models such as the assumption that every individual possesses an innate gender identity waiting to be discovered.

## 3 Reassessing the Evidence for Medical Intervention

During the early twenty‐first century, an international treatment model became established in which puberty suppression followed by cross‐sex hormone therapy came to be regarded as an important, and at times life‐saving, intervention for adolescents with gender dysphoria. These treatments were widely described as reversible and low‐risk. In 2015, for example, the Swedish National Board of Health and Welfare recommended that services ensure puberty blockers were initiated ‘without delay’, even in adolescents with only probable gender dysphoria, describing puberty suppression not only as a means of alleviating distress but as ‘a tool for diagnostically confirming gender dysphoria before decisions regarding cross‐sex hormone treatment’.

The National Board of Health and Welfare revised its recommendations following a systematic review by the Swedish Agency for Health Technology Assessment and Assessment of Social Services [3], which found insufficient evidence for the presumed benefits of these treatments alongside documented risks. Because the risks of puberty blockers were judged to outweigh their potential benefits, treatment should now be offered only in exceptional circumstances or within a research context. Several other European countries have reconsidered this treatment model on similar grounds. The most widely discussed reassessment is the Cass Review in England, which concluded that the evidence supporting medical intervention in children and adolescents with gender dysphoria was weak [4]. It also criticized the quality of clinical assessment, arguing that many young people had been channelled too quickly into a medical pathway rather than receiving holistic, multidisciplinary care.

A recent nationwide Finnish register study has attracted considerable attention [5]. It included every young person referred to specialist gender services in Finland between 1996 and 2019 and found that nearly half had received psychiatric care before their first assessment at a gender identity clinic. Use of specialized psychiatric services increased substantially among those who subsequently underwent hormone treatment, a finding difficult to reconcile with the claim that gender‐affirming medical treatment improves mental health. The study's strengths include nationwide coverage, complete follow‐up, objective register‐based outcomes rather than self‐report and considerably longer follow‐up than prior studies. As with any observational study, however, it cannot establish that gender dysphoria treatment itself caused the increase in psychiatric service use, and the underlying reasons for that increase remain unknown.

The debate continues. Proponents of early medical intervention emphasise the profound distress experienced by young people with gender dysphoria and argue that systematic evidence reviews underestimate its clinical benefits. Critics counter that these interventions may have irreversible consequences for fertility, sexual function and physical development in children and adolescents who may not yet have the maturity to appreciate their lifelong implications.

Both perspectives have merit. Gender incongruence can involve substantial psychological suffering and it is understandable that clinicians wish to relieve it. Some individuals with gender dysphoria benefit from medical and surgical intervention. At the same time, many young patients present with more complex clinical pictures and their long‐term prognosis remains uncertain. More research is needed to clarify the natural history of gender dysphoria in children, the extent to which treating co‐occurring psychiatric conditions may itself relieve distress, which adolescents are most and least likely to benefit from medical intervention and the long‐term consequences of hormone treatment. Until that evidence is available, the precautionary principle should prevail: *primum non nocere*.

## 4 Beyond Medicine: Sex, Self‐Declaration and Public Policy

This debate ultimately extends beyond medicine. Throughout human history, a person's sex has been understood to be identifiable from observable characteristics. In recent years, a growing number of countries have defined legal sex solely based on an individual's self‐declared identity rather than on characteristics observable by others. This shift in the legal basis for determining sex warrants scrutiny rather than uncritical acceptance. It raises the question of whether it is reasonable for public authorities, health services and commercial organizations to require deeply personal declarations of identity on official forms and in routine surveys. It also raises the question of whether it would not be preferable to retain biological sex as the primary category, with gender identity serving as a complementary concept for understanding individual experience.

## Author Contributions

**Mikael Landén:** conceptualization, writing – original draft, writing – review and editing.

## Funding

The author has nothing to report.

## Conflicts of Interest

The author declares no conflicts of interest.

## Linked Articles

This article is linked to Ludvigsson et al. and Ruuska et al. papers. To view these articles, visit https://doi.org/10.1111/apa.16791 and https://doi.org/10.1111/apa.70533.

## Data Availability

Data sharing not applicable to this article as no datasets were generated or analysed during the current study.
